# The effects of valproic acid on appetitive and aversive instrumental learning in adult rats

**DOI:** 10.3389/fnbeh.2014.00113

**Published:** 2014-04-01

**Authors:** John J. Orczyk, Melissa K. Banks, Preston E. Garraghty

**Affiliations:** ^1^Department of Psychological and Brain Sciences, Indiana UniversityBloomington, IN, USA; ^2^Program in Neuroscience, Indiana UniversityBloomington, IN, USA

**Keywords:** antiepileptic, anticonvulsant, depakote, valproic acid, cognition

## Abstract

Antiepileptic medications are the frontline treatment for seizure conditions. However, these medications are not without cognitive side effects. Previously, our laboratory reported learning deficits in phenytoin and carbamazepine-treated rats. In the experiment reported here, the effects of valproic acid (VPA) have been studied using the same instrumental training tasks. VPA-treated rats displayed a severe deficit in acquiring a tone-signaled avoidance response. This deficit was attenuated in animals that had prior training in an appetitive context. Thus, this deficit is specific to learning in an aversive context, and does not result from difficulties in transferring associations from an appetitive to aversive context. Learning transfer deficits were previously observed in rats treated with phenytoin, and to a lesser extent, carbamazepine. On the other hand, rats treated with VPA fail to suppress inappropriate responsiveness across aversive training whether they had undergone prior appetitive training or not.

## Introduction

Epilepsy is a class of neurological disorders characterized by reoccurring, unprovoked seizures (Chang and Lowenstein, 2003). Population estimates suggest that 0.5–7.1 in 1000 people are affected by epilepsy in any given year (Sander, 2003; Hirtz et al., 2007). Pharmacological intervention with antiepileptic drugs (AEDs) is the treatment of choice, but is not without cognitive side effects in animal models (Banks et al., 1999, 2001; Churchill et al., 2003; Samuelson et al., 2005; Umka et al., 2010) and human patients (Loring and Meador, 2001; Meador et al., 2003; Hamed, 2009). Our laboratory has previously shown that phenytoin, a commonly prescribed AED, blocked the acquisition of an avoidance response in the second part of an instrumental appetitive-to-aversive conditioning task (Banks et al., 1999). We have also shown that carbamazepine, another commonly prescribed AED, impairs the acquisition of an avoidance response and increases the number of inappropriate responses in the second part of the same appetitive-to-aversive conditioning task (Banks et al., 2001).

In the present study, we have extended our assessments to valproic acid (VPA), as it too is very commonly employed in treating seizure disorders. About 20% of epilepsy patients are treated with VPA (Hsieh and Huang, 2011). VPA is the most commonly prescribed AED in pediatric epilepsy cases, accounting for almost 40% of AED prescriptions (Kwong et al., 2012). VPA is also extensively used in the treatment of both manic and depressive phases of bipolar disorder (Nasrallah et al., 2006), and is considered a front-line treatment option for acute manic episodes (Singh and Berk, 2008). Since VPA is used to treat a variety of conditions, testing for side effects in healthy subjects is necessary to produce results that are relevant to all users. Additionally, testing in healthy subjects removes confounds arising from interactions between disorders and medication (Meador et al., 1995). Thus, any cognitive deficits observed with VPA treatment can be attributed to the drug and not to any disorder, or drug-by-disorder interaction.

The literature regarding the cognitive effects of VPA is mixed. There are reports in the human clinical literature suggesting that VPA exerts mild negative cognitive side effects. Patients treated with 250 mg per day of VPA for 1 month showed performance deficits relative to subjects treated with a placebo on delayed recall, digit span, and serial addition tests (Meador et al., 1995). Treatment with 1 g per day of VPA for 1 month also slowed decision making when subjects were asked to categorize objects by color or membership (Thompson and Trimble, 1981). Other studies report more severe cognitive disturbances associated with VPA treatment. One study reports severe cognitive dysfunction in 18 of 36 patients, accompanied by Parkinsonian symptoms such as tremor and bradykinesis. Importantly, the majority of cognitive and motor deficits were reversed upon termination of treatment with VPA (Armon et al., 1996) suggesting that the dysfunctions were associated directly with the drug and not with a neurological disorder *per se*. VPA treatment has also been shown to exacerbate cognitive decline when administered for HIV-related neuropathic pain in late stage HIV (Cysique et al., 2006).

Some studies using animal models have also reported cognitive side effects with VPA treatment, along with underlying neurological causes. Sub-chronic treatment with VPA increased the amount of time rats spent exploring objects in familiar locations on a novel object location test, suggesting a reduction in working spatial memory. VPA treatment reduced cell proliferation in the subgranular zone of the hippocampal dentate gyrus in the same rats (Umka et al., 2010). Treatment with 300 mg of VPA per kg produced a decrease in accuracy in a rat spatial delayed non-match-to-sample task when a delay of 4 s was used (Deacon, 1991). Cell proliferation in neuroblastoma and glioma cells have also been shown to be decreased in the presence of VPA in the therapeutic range (Regan, 1985).

Other studies, however, report an enhancement in the acquisition, extinction, and reinstatement of cued conditioned fear. Bredy and Barad (2008) report an increase in the freeze response after conditioned stimulus (CS) presentation in VPA-treated rats after 7 days of training. Moreover, VPA administration prior to partial fear-extinction trials significantly decreased the freezing response after 7 days of training in comparison to vehicle only treated rats. Additionally, VPA administration enhanced the reinstatement of conditioned fear 24 h after the presentation of a tone and shock in animals who previously underwent fear extinction conditioning. Enhancement of fear extinction was confirmed by Heinrichs et al. (2013) using C57BL/6 mice. VPA had no effect on extinction learning when a short duration CS was used, but significantly reduced the freezing response when a longer duration CS was used.

A few studies report improvements in performance on avoidance tasks after treatment with VPA. Treatment with sodium valproate improved the percentage of avoidance responses using a shuttle-box paradigm, while treatment with magnesium VPA had no effect. Only treatment with dipropylacetamide, a VPA derivative, had a negative impact on performance (Continella et al., 1984). Another study used a light/dark box in which rats were placed in the illuminated portion of the box and subjected to a foot-shock when entering the dark portion. Rats were administered scopolamine, an anticholinergic agent, in the 6 h following training. Scopolamine administration following avoidance training produces an amnesia-like effect. Treatment with pentyl-4-yn-VPA 3 h after training reduced the amnesia effect of scopolamine in a dose-dependent manner as demonstrated by longer latencies for entering the dark portion of the box when tested 24 h later (Murphy et al., 2001).

The amygdala has been found to underlie fear conditioning. In one study, bilateral lesions were made in the rat amygdala or dorsal hippocampus and underwent both cued and contextual fear conditioning. Contextual fear conditioning is multimodal and depends upon multiple background stimuli associated with a specific location, while cued fear conditioning only depends on a very discrete CS. Rats with lesioned amygdala failed to acquire the conditioned fear response in both cued and contextual paradigms. Rats with lesioned hippocampi showed no impairment in cued fear conditioning, but exhibited decreased performance in contextual fear conditioning. The results indicate that the hippocampus is involved in complex contextual fear conditioning, but not simpler cued fear conditioning (Phillips and LeDoux, 1992).

In the present study, we have employed a within-subject, tone-signaled bar press task in which rats are tested in both appetitive and aversive contexts. The task employed is complex and multi-contextual. There are multiple rules in the aversive context that must be learned through conditioning. In the aversive context, rats must learn to both press the lever after the tone, and not to press the lever during the inter-trial period. This paradigm was developed to study appetitive and aversive learning in the same subjects, and has been used in past work to evaluate learning, memory, and impairments that accompany cerebellar, hippocampal, cingulate, and prefrontal cortex lesions (Steinmetz et al., 1993; Logue, 1994). We have used this behavioral paradigm to evaluate the effects of phenytoin and carbamazapine in adult rats (Banks et al., 1999, 2001; McDowell et al., 2004), rats exposed to phenytoin in utero (Mowery et al., 2008), rats with lesions of the basal nucleus of Meynart (Butt et al., 2003), ovariectomized female rats with or without estradiol replacement (Goodman et al., 2004), and rats undergoing chronic restraint (McDowell et al., 2013).

Although previous studies have shown VPA to enhance the extinction of fear conditioning (Bredy and Barad, 2008; Heinrichs et al., 2013) or performance on a simple avoidance task (Continella et al., 1984), they have also shown VPA to be detrimental to spatial learning in association with decreased cellular proliferation in the hippocampus (Umka et al., 2010). The complex nature of the aversive task employed in this study would suggest that the hippocampus facilitates task learning. Therefore, it is hypothesized that VPA will interfere with the acquisition of the avoidance response in the aversive context due to detrimental effects of VPA on the hippocampus.

## Materials and methods

Adult Sprague-Dawley rats bread in the Indiana University animal care facilities were used. For comparison with previous studies (Banks et al., 1999, 2001) and to exclude sex as a confounding variable, only female rats were tested. In the literature, however, there is no indication in that cognitive deficits associated with AEDs are gender-linked as deficits are expressed in both males and females (Meador et al., 1995). The estrus cycle was not monitored as subject participation in the experiment extended over roughly 60 days, and underwent multiple cycles. Any within-subject variability possibly arising from hormonal fluctuations would therefore be expected to be averaged out over the extended training period.

The animals were maintained at 85% free-feeding body weight throughout the study. The 45-mg food pellets used as appetitive reinforcers (Bio-Serve, Frenchtown, NJ) were introduced to the animals in the home cage at least 2 days prior to the beginning of training. Animals were tested in an operant chamber (Lafayette) placed in a lighted (10-W utility bulb) sound-attenuating chamber, which contained a center-mounted speaker to deliver the tone (2 kHz at 90 dB sound pressure level (SPL)). The front wall of the operant box consisted of one bar at center and a recessed food well on the left.

### Back wire implantation

All animals underwent a simple surgical procedure to implant two back wires, necessary as the connection point for the active lead during the aversive component of the task, before beginning shaping and training for the first (or only) component. Animals were anesthetized with a mixture of ketamine and xylazine (60 and 6 mg/kg, respectively, intramuscular (IM)), with supplemental doses given as needed. Two double-loop wires (30 gauge surgical), approximately 1 cm apart, were threaded subcutaneously between the scapulae of each animal. Animals also received 0.2 cc Dopram (IM) and triple antibiotic ointment on the area of the wires. The entire procedure took approximately 15 min per subject.

### Drug administration

VPA (Depakote; Sigma-Aldrich, St. Louis, MO) was administered as stated in the detailed methods for each experimental condition. The VPA oral suspension (125 mg/5 ml) was administered to each animal via gavage twice daily. Oral administration was used in rats in order to maintain consistency with the route of administration most commonly used in humans. VPA treated animals received 150 mg of the drug per 1 kg body mass. One of the two daily dosages was delivered 2 h before training. This regimen has been previously shown to produce plasma levels within the human therapeutic range during the training period (Churchill et al., 2003).

### Appetitive-to-aversive transfer task

#### Appetitive context

All sessions, appetitive or aversive, were separated by 24 h. In the appetitive context, the animals were first shaped using a method of successive approximations to bar-pressing behavior for food reinforcement. When the animal pressed the bar 100 times in 30 min on a continuous reinforcement schedule, they were advanced to a fixed-ratio reinforcement schedule (FR4) to strengthen the behavior (i.e., render it more resistant to extinction). They were then required to perform 400 bar presses (receiving 100 pellets) within 30 min on two consecutive days before they began the tone training trials.

During the tone-signaled sessions, only a response during the tone period was reinforced. One session consisted of 100 tones, each lasting 3 s or until the food pellet was delivered, followed by a 15 s intertrial interval (ITI) and a randomly determined 1–8 s pretone period. If the rats pressed the bar during the pretone period, the period was reset and the trial was delayed until no bar presses occurred during the randomly determined pretone period. Animals continued appetitive tone training for a total of 31 days.

#### Aversive context

The rats were then transferred to a bar-press avoidance task. The aversive context consisted of a shock that could be terminated by a bar press. The shock intensity was usually maintained at 0.7 mA. If the animal did not respond to the shock level, it was increased slightly until a level was found at which the animals responded consistently, but never to exceed 1.0 mA. The animals were introduced to the aversive context in a single training session where the shock pulses were presented continuously until the bar was pressed. If the animal did not press the bar, a rest period of 30 s was initiated. Subjects were required to press the bar prior to the onset of the fifth shock pulse at least 15–20 times consecutively, and were advanced to tone trials on the next session day. The tone was the same used in the appetitive conditioning (2 kHz, 90 dB SPL). On tone-signaled trials, the impending foot shocks could be avoided by a bar press during the first 3 s of tone presentation, or escaped by a bar press in the latter 3 s after the shock was initiated. The shock was delivered as a series of four 250 ms pulses separated by 500 ms periods of no shock. To prevent the animals from adopting a strategy of holding the bar down for excessive amounts of time (thereby avoiding the shock), continuous shock pulses were delivered if the animal failed to release the bar after 5 s. These shocks are termed off-bar shocks (OBS). The trials were separated by 8–12 s ITIs and a variable 2–6 s pretone period. A bar press during the ITI or pretone period reset the pretone period and delayed the initiation of the next trial. One session of avoidance learning consisted of 300 tone presentations, or 300 chances to avoid or escape the shock. The aversive phase of the experiment continued for 25 days.

### Experimental conditions

#### Effects of VPA on appetitive-to-aversive transfer

Separate cohorts received treatment with VPA (*N* = 7) or water only (*N* = 6) at the conclusion of the 21st day of appetitive training. These animals continued appetitive tone training for 10 days to assess the effect of the drug and/or gavage procedure, if any, on the acquired tone-signaled bar press. In addition, a third cohort of rats served as controls (*N* = 12), receiving no gavage treatment throughout the appetitive and avoidance training. After initiation of drug or water treatment, behavioral testing began 2 h after VPA or water administration. Drug or water administration continued daily throughout the remaining appetitive and total number of avoidance training sessions.

#### Effects of VPA on avoidance acquisition without prior appetitive experience

**Figure 1:**
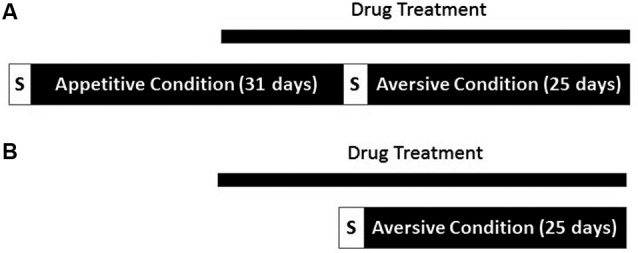
**Overview of experimental procedure. (A)** One group of rats underwent both appetitive and aversive conditioning. Drug administration began 21 days into conditioning in the appetitive context. Both contexts were proceeded by a brief period in which responses were shaped (S). **(B)** A separate cohort of rats underwent conditioning in the aversive context only. Drug treatment was initiated 11 days before the start of conditioning in the aversive context.

Another group of animals were treated with VPA (*N* = 9) or water (*N* = 10) treatment was initiated, and continued for 10 days, at which time the animals began avoidance training. Animals performed one session of aversive shaping and began the 25 days of tone-signaled avoidance training the following day. All parameters in the aversive context remained as described above, but without prior exposure to the appetitive context. An overview of the two experimental conditions appears in Figure 1.

### Calculations and statistics

In the appetitive context, the correct response rate (CR) is calculated as the percentage of reinforced bar presses out of the total number of trials. In the aversive context, the CR is calculated as the percentage of successful avoidance responses out of the total number of trials. In both contexts, efficiency ratios (ERs) are calculated by dividing the number of CRs by the total number of bar presses. Percent positive transfer measures the impact of prior appetitive experience on performance in the aversive context. Percent positive transfer is calculated in terms of aversive CRs by:

$$\frac{100\times {\text{CR}}_{\text{with appetitive experience}}}{{\text{CR}}_{\text{with appetitive experience}}}-100$$

Percent positive transfer is also calculated in terms of aversive ERs by replacing CRs with respective ERs. Positive values indicate a beneficial effect of prior appetitive experience on aversive performance while negative values indicate a detrimental effect of prior appetitive experience on aversive performance.

To evaluate differences in performance between the drug-treated and control groups across training days, a mixed-design (split-plot) repeated-measure analysis of variance model was applied (ANOVA). Statistical decisions were based on a 0.05 significance level. The calculations were carried out using SPSS 19.0 software, on a computer running Microsoft Windows 7.

## Results

**Figure 2:**
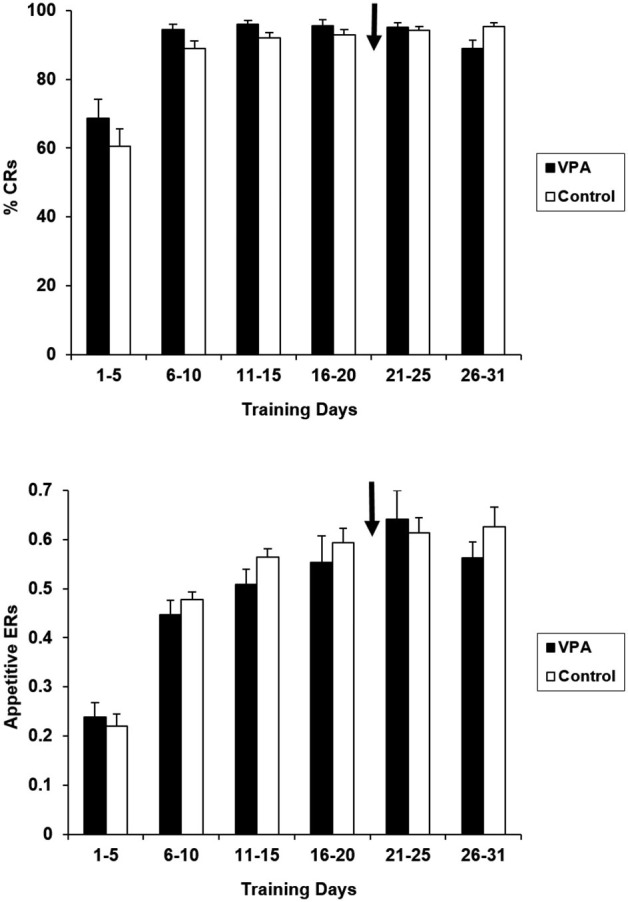
**Appetitive percent reinforced responses (top) and efficiency ratios (bottom) for control and VPA treated animals are displayed**. Percentage of reinforced bar presses correct response rates (%CRs) and efficiency ratios (ERs) for control animals demonstrate that these animals learn the appetitive task. Arrow denotes beginning of drug treatment. Since drug treatment was initiated on the 21st day of appetitive training, any difference in acquisition on days 1–20 is due to individual differences. Valproic acid (VPA)-treated animals show no negative effect on the maintenance of appetitive performance. Error bars display the standard error of the mean.

VPA treatment had no impact on the performance in the appetitive context. Figure 2 presents percentages of reinforced bar presses (CRs) and ERs (reinforced bar presses/total number of bar presses) for VPA-treated and untreated controls. Water-treated and untreated control groups have been collapsed together for presentation because they did not differ for any of the training days [*F*_(1,16)_ = 2.203, *p* > 0.05]. As expected, during the first 5 days of training there were no statistically significant differences between the VPA-treated and control groups in terms of CRs [*F*_(1, 17)_ = 1.092, *p* > 0.05], or ERs [*F*_(1, 17)_ = 0.212, *p* > 0.05]. After drug treatment was initiated at the conclusion of the 21st day of training, performance remained indistinguishable between control and drug treatment groups. For the final 5 days of appetitive training, neither CRs [*F*_(1, 17)_ = 0.165, *p* > 0.05], nor ERs [*F*_(1, 17)_ = 0.086, *p* > 0.05], were significantly different. Likewise, CRs, [*F*_(1, 17)_ = 1.419, *p* > 0.05] and ERs and [*F*_(1, 17)_ = 0.151, *p* > 0.05] remained comparable over the entire 31 days course of appetitive training. Response latencies were also measured for appetitive trials. As expected, the average response latency of the group of rats treated with VPA remained stable before initiation of drug treatment (924.6 ± 94.2 ms) and at the conclusion of appetitive training [888.7 ± 76.8 ms; *t*_(12)_ = 3.15, *p* > 0.05].

**Figure 3:**
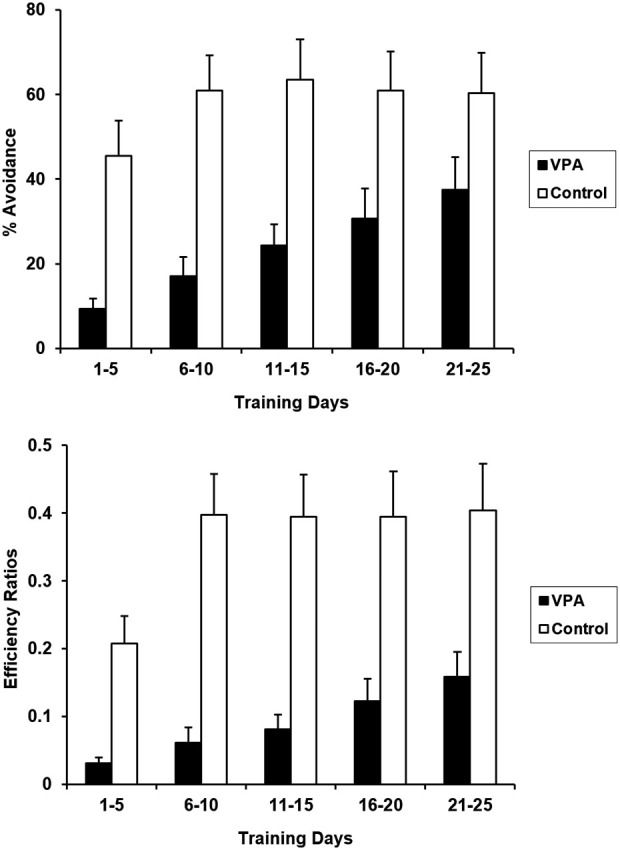
**Aversive percent avoidances responses (top) and efficiency ratios (bottom) for control and VPA treated animals, without prior training in the appetitive context, are displayed**. VPA-treated animals displayed drastically reduced acquisition and expression of the avoidance response, in comparison to control animals. Prior experience in an appetitive context was advantageous for VPA-treated animals, but had not effect for controls. Error bars display the standard error of the mean.

Rats undergoing aversive conditioning without prior appetitive experience were treated with VPA 10 days before the training session began. VPA treatment significantly impaired acquisition and expression of the avoidance response in the absence of appetitive experience (Figure 3). CRs for the entire 25 days training period differed significantly for control and VPA-treated rats [*F*_(1, 20)_ = 9.200, *p* < 0.05]. Likewise, ERs were also significantly different [*F*_(1, 20)_ = 13.825, *p* < 0.05]. VPA-treated animals showed severe deficits in both the acquisition and terminal expression of the avoidance response. During acquisition (days 1–5), control animals exhibited a daily average CR of 45.5% while VPA-treated animals exhibited an average CR of only 9.4%. Terminal expression (days 21–25) of the avoidance response was also negatively affected by VPA, with controls exhibiting an average CR of 60.4% while VPA treated animals only exhibited an average CR of 37.4%. Lower ERs for VPA-treated rats indicates that their bar pressing behavior is more random than controls.

**Figure 4:**
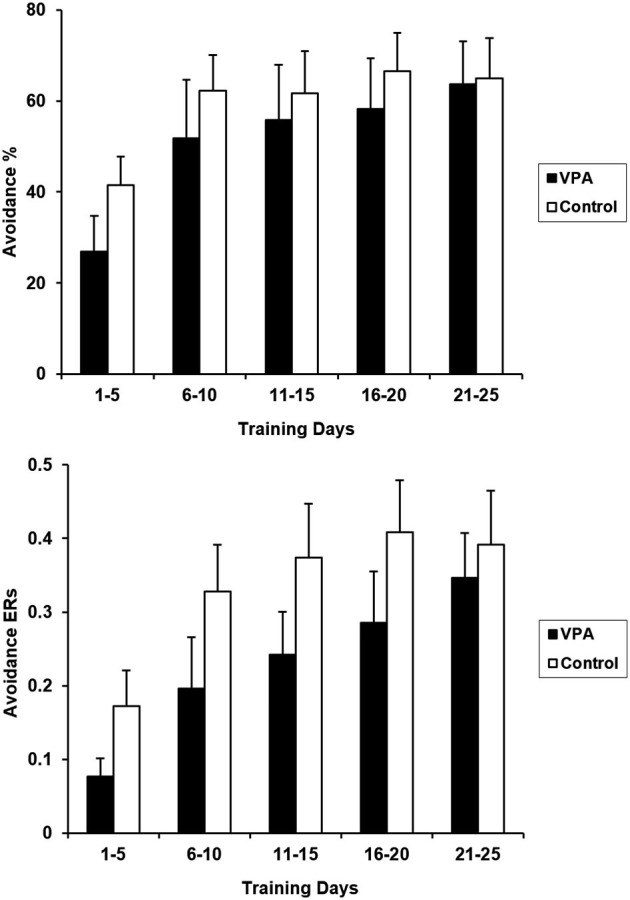
**Aversive CRs (top) and ERs (bottom) for control and VPA treated animals, following training in the appetitive context, are displayed**. Although the acquisition of the avoidance response appears to be delayed in VPA-treated animals, differences were found to be statistically insignificant (see text). The terminal expression of avoidance behavior for both groups is comparable, demonstrating that VPA did not significantly affect appetitive-to-aversive transfer. Error bars display the standard error of the mean.

Prior appetitive experience attenuated performance deficits in VPA-treated rats. Figure 4 presents CRs and ERs for the entire course of avoidance training for VPA-treated and control rats. Water-treated and untreated controls were again collapsed in this figure since avoidance performance is comparable between these two groups in terms of both CRs [*F*_(1,15)_ = 0.003, *p* > 0.05] and ERs [*F*_(1,15)_ = 0.002, *p* > 0.05]. VPA treatment had no significant effect on appetitive to aversive transfer as CRs over the 25 days of training were not statistically different [*F*_(1, 17)_ = 0.402, *p* > 0.05]. Similarly, control and VPA-treated ERs were also not statistically different from each other when compared across the entire training period [*F*_(1, 17)_ = 0.997, *p* > 0.05].

Aversive learning in VPA-treated animals varied substantially depending on whether there had been prior appetitive training. To further underscore this difference, avoidance learning behavior was compared directly in the presence or absence of prior appetitive experience. The daily average avoidance rate over the first 5 days of training for the VPA-treated animals with prior appetitive training was 26.9% as compared to just 9.4% for the animals that had no previous appetitive training. Over the last 5 days of training, the daily average avoidance rates were 63.6% and 37.4% respectively for the VPA-treated rats with or without prior appetitive training. Comparing performance across the entire training period, the two groups differed significantly [*F*_(1, 14)_ = 6.444, *p* < 0.05]. A similar pattern is present for the ERs. Over the first 5 days of training, the daily average ER for the animals with prior appetitive experience was 0.080 as compared to 0.031 for those without. Over the last 5 days of training the averages were 0.359 and 0.158 respectively. A repeated measures ANOVA comparing performance across all training days found the two groups to differ significantly [*F*_(1, 14)_ = 7.940, *p* < 0.05].

**Figure 5:**
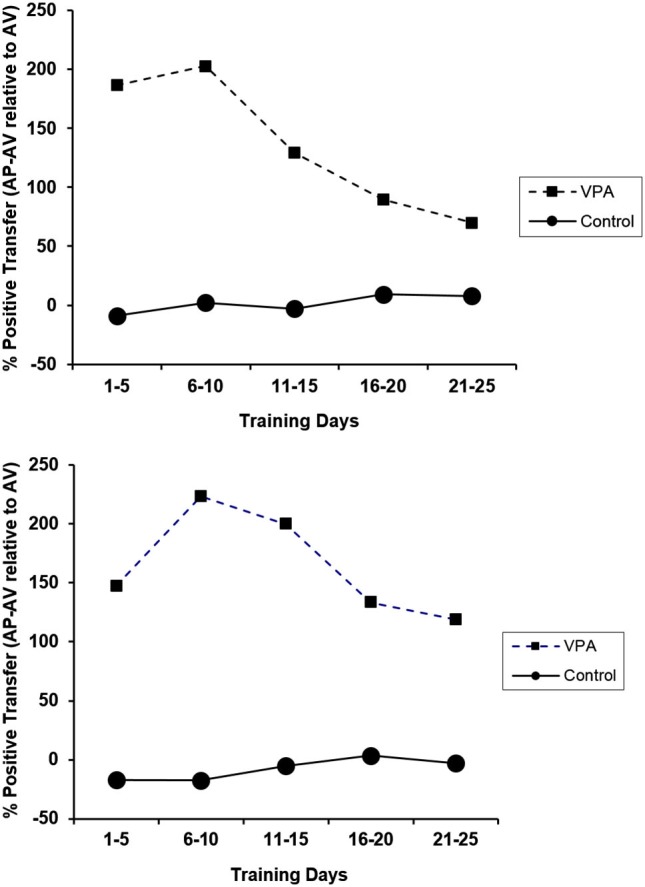
**Percent positive transfer (prior appetitive experience relative to aversive experience only) based upon percent avoidance response (top) and ERs (bottom) for control and VPA-treated animals**. Prior appetitive experience greatly enhanced both avoidance rates and ERs for VPA-treated animals. Interestingly, prior appetitive experience did not affect the performance of drug-naïve animals in either direction. Error bars display the standard error of the mean.

The percent positive transfer for CRs and ERs of animals with prior appetitive experience as compared to animals placed directly into the aversive context is presented in Figure 5. Control animals performed similarly in the presence or absence of prior appetitive experience. VPA-treated animals, on the other hand, profited substantially from appetitive training prior to the avoidance learning challenge. This high degree of learning transfer from the appetitive to aversive context underscores the remarkably important contribution of appetitive training to avoidance learning in VPA-treated animals. The figure also illustrates that the performance deficit observed when placed directly into the aversive learning context does not reflect a drug-related problem with avoidance learning *per se*. The deficit is more nuanced as VPA-treated rats with prior appetitive experience performed sufficiently well in the aversive context such that performance was not significantly different from controls.

**Figure 6:**
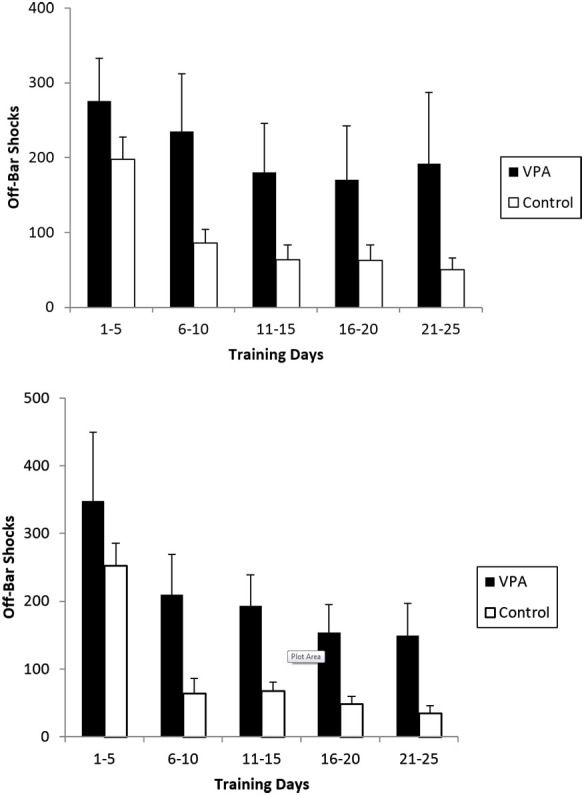
**The average number of off-bar shocks (OBS) received during an aversive training session for rats exposed to both the appetitive and aversive contexts (top) and those exposed to the aversive context only (bottom)**. Repeated measures ANOVA demonstrated the difference to be significant across all 25 individual training days. In both cases, VPA-treated animals failed to suppress the primitive response of pressing and holding the bar, while controls abandoned this strategy in the first 5 training sessions. Error bars display the standard error of the mean.

OBS are applied when an animal fails to release the bar after 5 s in the aversive context and are a measure of inappropriate responses. VPA-treated animals with prior appetitive experience received an average of 275 OBS while controls only received 198 (Figure 6, **top**). The difference is more striking for the final 5 days of training were VPA-treated animals still received an average of 192 OBS while control animals only received an average of 50. A mixed-design repeated measures ANOVA across all 25 training days revealed the difference in OBS received to be significant [*F*_(1, 20)_ = 8.853, *p* < 0.01]. VPA-treated animals lacking prior appetitive experience also incurred substantially more OBS than control animals (Figure 6, **bottom**). During the first 5 training days, both VPA-treated and control animals experienced more OBS on average than their counterparts who had appetitive training, with the groups receiving on average 348 and 252 OBS respectively. During the final 5 training days, control animals reduced the average number of OBS received to 39 while VPA treated animals received an average of 149. The difference across all 25 training days was found to be statistically significant [*F*_(1, 20)_ = 4.520, *p* < 0.05].

## Discussion

Conflicting reports of cognitive deficits in patients maintained on AEDs have motivated our ongoing evaluation of these drugs using a variety of behavioral paradigms (Churchill et al., 1998, 2003; Banks et al., 1999, 2001; McDowell et al., 2004; Samuelson et al., 2005). The present study expands our efforts to VPA, using the same well-defined instrumental learning and memory paradigm used to investigate phenytoin and carbamazepine in previous studies. The results demonstrate drug-specific cognitive deficits, with VPA inhibiting the acquisition of the avoidance response in the absence of prior appetitive training. Unlike our previous study where phenytoin impaired the transfer between appetitive and aversive contexts (Banks et al., 1999), prior appetitive training enhanced acquisition of the avoidance response in VPA-treated animals relative to animals placed directly into the aversive learning context.

VPA treatment had no apparent effect on the animals’ already-acquired appetitive performance. VPA treatment was initiated on the 21st day of appetitive conditioning and maintained throughout the remaining appetitive and aversive training. We find no differences in appetitive performance of the VPA-treated animals when comparing pre- (training days 16–21) to post-drug (training days 26–31) data, with both reinforced responses and ERs remaining stable and comparable to controls (Figure 2). Response latencies were shown to remain stable between the initiation of drug and the conclusion of appetitive training. The effects of VPA on instrumental performance are not likely attributable to sensorimotor disruptions. A slowing of sensory processing of the cue stimulus and/or response production could generate an apparent avoidance learning deficit if the animal was not able to produce the response within the initial 3 s tone period. The absence of any response latency difference following the onset of drug treatment clearly suggests that neither of these performance factors suffered from the drug treatment.

VPA treatment diminished aversive performance in rats without prior appetitive experience. Whereas control animals reached asymptotic performance within the first 10 days of training, the performance of VPA-treated animals was substantially reduced throughout training (Figure 3). An even greater difference is observed in terminal ERs. Over the last 5 days of avoidance training, the VPA-treated animals were making in excess of 7.5 bar presses for each successful avoidance, whereas control animals were making less than 3 bar presses per avoidance. While control animals did produce fewer bar presses than VPA-treated animals on average (557 and 878 bar presses per session, respectively, across the last 5 days of training), this difference is not large enough to fully account for the difference in ERs. Rather, the control animals were clearly distributing their responses more appropriately with respect to the tone signal than were the drug-treated animals.

Prior appetitive experience attenuated aversive performance deficits in VPA-treated rats. Following the conclusion of appetitive training, the rats were transferred to an aversive context in which an aversive stimulus could be actively avoided with a tone-signaled bar press (Figure 4). At first appearance, VPA-treated animals appear to have a slight delay in avoidance acquisition, with terminal performance reaching levels comparable to controls. However, a repeated measures ANOVA failed to reveal any significant difference between the average CRs or ERs for VPA-treated and control animals for the entire course of training. Any possible performance difference between VPA-treated and control animals is relatively mild and diminishes early in training, at least in terms of CRs and ERs.

OBS are administered to animals that maintain a sustained bar press irrespective of the stimulus conditions. Behavior resulting in OBS can be characterized as “primitive” avoidance responses, indicating the learning of some fundamental relationship between the bar presses and shock cessation. Regardless of prior appetitive experience, VPA-treated animals experienced more OBSs throughout the 25 days aversive context, suggesting some degree of cognitive impairment. Both control and VPA-treated animals begin aversive training with a relatively high number of OBS. By the end of the 25 days of training, control animals have generally learned to suppress this primitive response while VPA-treated animals show relatively little decline in this inappropriate and ultimately ineffectual behavior. The larger number of OBS received by VPA-treated animals during all 25 days of the aversive-only context contributed to lower ERs. Even in the absence of prior appetitive training, control animals learned to suppress the primitive avoidance response while VPA-treated animals failed to appropriately distribute bar presses (Figure 6, **bottom**). This failure of VPA-treated animals represents a learning deficit in an aversive context, regardless of prior appetitive experience.

In the aversive condition, rats must learn at least two rules: press the bar directly after the tone/light signal, and avoid pressing the bar during the ITI. Failure to follow either one of these rules results in electrical shock. Moreover, the former rule requires pressing the lever while the later rule requires not pressing the lever. Difficulty in lever pressing and avoiding electrical shocks may arise due to the complexity of the task. It is possible that learning multiple tasks in the aversive context was undermined by VPA. The high rate of OBS received by both groups of VPA-treated animals indicates that neither group fully learned not to press the bar in the ITI and avoid the resulting OBS. Apparently without the benefit of appetitive training, VPA-treated animals also failed to fully learn the tone/light signal and response of pressing the bar to avoid electrical shock. Nonetheless, both control animals and VPA-treated animals that had the benefit of appetitive experience managed to avoid the tone/light signaled shocks at a greater frequency that did VPA-treated animals lacking appetitive experience.

VPA has been shown to strengthen fear-conditioning (Bredy and Barad, 2008), extinction of conditioned fear (Bredy and Barad, 2008; Heinrichs et al., 2013), and reinstatement of cue-induced appetitive operant conditioning (Ploense et al., 2013). VPA decreases cellular proliferation while putatively promoting cellular transcription through preventing chromatin compacting associated with deacetylation of lysine residues (Phiel et al., 2001). Different memory mechanisms are involved in Pavlovian conditioning, cue-induced fear-conditioning, and contextual fear-conditioning. Cue-induced fear-conditioning requires activation of the amygdala. Context-induced fear conditioning, however, requires activation of both the amygdala and the hippocampus (Phillips and LeDoux, 1992). Inhibiting hippocampal adult neurogenesis has been shown to impair acquisition of a conditioned-fear response using a trace conditioning paradigm, a hippocampal-dependent task (Shors et al., 2002). VPA has been previously shown to inhibit hippocampal neurogenesis (Umka et al., 2010). Perhaps this effect of VPA is detrimental to acquiring the avoidance response without prior appetitive experience.

As with VPA, we have previously reported that treatment with phenytoin did not affect the maintenance of the appetitive response. In contrast to VPA, phenytoin-treated animals did not display an avoidance learning deficit when placed directly into the aversive context. In the absence of prior appetitive experience, phenytoin-treated animals performed as well as controls in terms of CRs and ERs. Also in contrast to VPA-treated rats, prior appetitive experience had a detrimental effect on avoidance acquisition in phenytoin-treated animals. In these animals, prior appetitive experience interfered with learning. Phenytoin-treated animals consistently displayed CRs of 20% or less and ERs of 0.1 or less across all 25 training days in the presence of prior appetitive training (Banks et al., 1999).

As with VPA and phenytoin, carbamazepine also failed to affect the maintenance of the appetitive response (Banks et al., 2001). Carbamazepine increased the variability of CR and ER performance, compared to controls, suggesting that the effects of carbamazepine vary greatly within groups. Carbamazepine-treated animals also failed to suppress the primitive avoidance response, leading to numbers of OBS comparable to VPA treated animals following appetitive training. Unlike VPA-treated animals, however, no differences were observed between carbamazepine treated animals and controls is the absence of prior appetitive training (Banks et al., 2001). Thus, carbamazepine causes a slight impairment in learning transfer from appetitive to aversive learning, while VPA does not.

A major limitation of this study is the sole reliance on behavioral data and lack of neurological evidence. Although other studies report that VPA decreases hippocampal adult neurogenesis (Umka et al., 2010), effects of VPA on hippocampal neuronal proliferation were not directly tested in this study. Future studies on the cognitive effects of VPA should compare cue-induced with contextual fear conditioning, and utilize histological methods in addition to behavioral methods. Moreover, additional work remains to be performed in order to delineate VPA impairment of acquisition of the tone-barpress association from its expression. This could be suitably studied by initiating drug treatment 11 days into the aversive context, by which time the tone-barpress associated would have already been acquired.

The three first-generation AEDs studied thus far show different types and severities of cognitive impairment. Phenytoin is associated with severe impairment in the transfer of learning from the appetitive to aversive context, to the point where prior appetitive experience interferes with acquisition of even the primitive avoidance response. However phenytoin does not affect learning in an aversive context per se as phenytoin-treated animals lacking prior appetitive experience display no performance deficits (Banks et al., 1999). The detrimental effects of carbamazepine seem to be a milder impairment than that observed with phenytoin treatment. Carbamazepine-treated animals readily acquired the primitive avoidance response, but failed to suppress it following appetitive training. Since this deficit was not observed in the absence of prior appetitive training, it is also not a learning deficit in aversive context *per se*, but indicates impairment in learning transfer as well (Banks et al., 2001).

Cognitive impairment associated with VPA is fundamentally different from phenytoin or carbamazepine. Prior appetitive experience greatly facilitated the acquisition of the proper avoidance response, to the extent that CRs and ERs are not significantly different from control animals. However, the larger number of OBS suggests that VPA-treated animals were not nearly as capable of suppressing this response as control animals. In the absence of prior appetitive training, significantly different CRs and ERs, in addition to a greater number of incurred OBS, demonstrate impaired learning in aversive contexts. Presumably, second-generation AEDs will provide greater efficacy with less detrimental side effects. Future research will extend our analysis to these drugs using the same learning transfer paradigm.

## Conflict of interest statement

The authors declare that the research was conducted in the absence of any commercial or financial relationships that could be construed as a potential conflict of interest.
